# Understanding the Bacterial Response to Mycotoxins: The Transcriptomic Analysis of Deoxynivalenol-Induced Changes in *Devosia mutans* 17-2-E-8

**DOI:** 10.3389/fphar.2019.01098

**Published:** 2019-11-14

**Authors:** Yousef I. Hassan, Jian Wei He, Dion Lepp, Ting Zhou

**Affiliations:** Guelph Research and Development Centre, Agriculture and Agri-Food Canada, Guelph, ON, Canada

**Keywords:** Deoxynivalenol, *Devosia*, RNA_Seq, prokaryotes, detoxification

## Abstract

Deoxynivalenol (DON) is a major fusarium toxin widely detected in cereal grains. The inadvertent exposure to this fungal secondary-metabolite gives rise to a myriad of adverse health effects including appetite loss, emesis, and suppression of the immune system. While most of the attention this mycotoxin has gained in the past four decades was related to its eukaryotic toxicity (monogastric animals and plants more precisely), recent studies have begun to reveal its negative influence on prokaryotes. Recently presented evidence indicates that DON can negatively affect many bacterial species, raising the possibility of DON-induced imbalances within the microbiota of the human and animal gut, in addition to other environmental niches. This in turn has led to a greater interest in understanding bacterial responses toward DON, and the involved mechanism(s) and metabolic pathways, in order to build a more comprehensive picture of DON-induced changes in both prokaryotes and eukaryotes alike. This study reveals the transcriptomic profiling of *Devosia mutans* strain 17-2-E-8 after the inclusion of DON within its growth medium. The results highlight three adaptive mechanisms involved in the response of *D. mutans* 17-2-E-8 to this mycotoxin, which include: (a) activation of adenosine 5’-triphosphate-binding cassette transporters; (b) engagement of a toxin-specific pyrroloquinoline quinone-dependent detoxification pathway; and finally (c) the upregulation of auxiliary coping proteins such as porins, glutathione S-transferases, and phosphotransferases. Some of the identified mechanisms are universal in nature and are shared with other bacterial genera and species.

## Introduction

Deoxynivalenol (DON) is a major fusarium mycotoxin widely present in cereal food/feed, which is produced by invasive fungal species during plant pathogenesis. Exposure of humans and animals to this fungal secondary-metabolite manifests in a myriad of short- and long-term adverse health effects, ranging from appetite loss, emesis, decreased weight gain, to the suppression of immune systems, depending on the exposure dose, its frequency, and the physiological stage of the exposed host (Hassan et al., 2015). Moreover, the ability of DON to interfere with the absorption of many nutrients, through the suppression of protein biosynthesis (Peng et al., 2017a) and/or interference with cellular membrane integrity (De Walle et al., 2010; Ghareeb et al., 2015), is well documented.

The past decade has witnessed a vast increase in our knowledge of gut microbiota and its role in human and animal health, which has led to greater scrutiny of environmental factors that may influence its composition. As most bacterial hosts can tolerate many-fold higher concentrations of DON than eukaryotes (Takakura et al., 2014), the toxicological effects of DON on most prokaryotes have been considered erroneously negligible (Suzuki and Iwahashi, 2015). However, several recent reports have revealed that exposure to DON can affect intestinal mucus production and the functional diversity of gut microbiota (Piotrowska et al., 2014; Peng et al., 2017b; Robert et al., 2017), as well as increasing the genotoxicity of certain chemicals in specific hosts (Payros et al., 2017), thereby highlighting the need for further investigations of DON effects in prokaryotes as well as in eukaryotes (Park et al., 2014; Ishikawa et al., 2017). A better understanding of the bacterial response to DON at the transcriptome level could also lead to the identification of more novel bacterial detoxification mechanisms, which can be applied to help address the growing problem of DON contamination in agricultural commodities.

The present work reports on the transcriptional response of *Devosia mutans* 17-2-E-8, an efficient DON detoxifier, to DON exposure probed by transcriptome profiling and connecting the observed changes with phenotype outcomes.

## Materials and Methods

### Chemicals, Media Formulations, and Bacterial Cultures

*D. mutans* 17-2-E-8 cells were grown and maintained as reported previously (He et al., 2016). The high-performance liquid chromatography (HPLC)-grade methanol used for extractions was obtained from Caledon Labs (Georgetown, ON, Canada), while DON was obtained either from Sigma-Aldrich (Oakville, ON, Canada) or TripleBond (Guelph, ON, Canada). Bacterial pellets were collected by centrifugation at 8,500 rpm for 30 min and stored frozen at -20°C until usage unless stated otherwise. 3-*epi*-DON, used as standard, was prepared as reported previously (He et al., 2015b).

### Effect of Deoxynivalenol on *D. mutans* 17-2-E-8 Growth and Deoxynivalenol Epimerization

In order to establish the inhibitory effect of DON on the growth of *D. mutans* 17-2-E-8 cells and their DON epimerization capacity, cultures (1.5 ml) of *D. mutans* 17-2-E-8 containing 1 × 10^6^ colony forming units per milliliter were added as inoculum to cornmeal broth (He et al., 2016) (12.0 ml) supplemented with DON standards (1.5 ml) in sterile water. The final concentrations of DON spanned the 100–4,000 μg/ml range. These cultures were incubated at 28°C on a rotary shaker at 200 rpm up to 132 h. Bacterial cell numbers were enumerated every 12 h by plating serial dilutions onto cornmeal agar plates. The number of colony forming units of each dilution was determined after 72–96 h incubation at 28°C.

To evaluate the effect of DON on the ability of *D. mutans* 17-2-E-8 to enzymatically transform DON into 3-*epi*-DON (He et al., 2016), 150 μl of the liquid bacterial culture was collected at 6, 12, 24, 36, 48, 60, 72, 84, 96, 108, 120, and 132 h. The reactions were stopped by adding 150-μl methanol. Mixtures were allowed to stand for 2 h then centrifuged at 18,000×*g* for 5 min (Micromax^®^ Microcentrifuge, Milford, MA, USA) before analyzing DON by the HPLC approach as outlined previously (He et al., 2016). An Agilent Technologies 1100 Series HPLC system equipped with a Luna C18 column (150 × 4.6 mm, 5 μm) (Phenomenex, Torrance, CA, USA) was used in the previously stated analyses. The binary mobile phase consisted of methanol (solvent A) and water (solvent B), and the gradient program started at 22% A, increased linearly to 41% A at 5 min, 100% A at 7 min, held at 100% A from 7 to 9 min, and returned to 22% A at 11 min. There was a post-run column reconditioning phase (2 min) under the starting conditions. The flow rate was held constant at 1.0 ml/min, and the detector was set at 218 nm. The identification and quantification of DON and *3-epi*-DON were achieved through comparing the retention times, ultraviolet–visible spectra, and concentrations of pure standards. Bacterial growth and DON transformations were conducted in triplicate, and the mean values were reported.

### Challenging *D. mutans* 17-2-E-8 With Deoxynivalenol

An overnight *D. mutans* 17-2-E-8 culture grown in 60-ml lysogeny broth (supplemented with 50 μg/ml kanamycin) at OD_600_ > 1 was challenged with DON at a final concentration of 50 µg/ml. Samples (10 ml each) were collected before DON addition and at 1, 3, and 5 h after DON addition. Cells were harvested by centrifugation for 5–10 min at 13,000 rpm, and RNA*later* Solution (Ambion, cat. # AM7021) was added for RNA stabilization during the cold storage.

### Total RNA Preparation and Transcriptomic Profiling

Total RNA extractions were conducted using the RNeasy Mini kit (Qiagen, Toronto, ON; cat. #74104) according to the manufacturer’s recommendations. RNA integrity and concentrations were evaluated using the Bio-Rad Experion Automated Electrophoresis System (Bio-Rad Laboratories, Mississauga, ON). After ensuring the quality and integrity of the obtained RNA samples (**Supplementary Figures 1A, B**), the samples were treated with rDNase I (Ambion) for 1 h at 37°C (cat #: AM2235, Life Technologies Inc.) following the manufacturer’s instructions.

rRNA depletion was performed using RiboMinus Transcriptome Isolation Kit—bacteria module (Thermo Fisher Scientific, Ottawa, ON; cat. # K155004) according to the recommended protocol, and RNA-Seq libraries were prepared from 200 ng of RNA at the University Health Network/Mount Sinai Hospital—Gene Profiling Facility (Toronto, ON). The validated libraries were pooled and sequenced on HiSeq 2500 Sequencing System (Illumina, Inc.) with 100-bp paired-end sequencing runs. The resulting reads were analyzed using CLC Genomics Workbench 6.5.2 (CLC, http://www.clcbio.com/products/clc-genomics-workbench/) according to the supplier’s procedure. In essence, a transcriptomic analysis was initiated with unpaired two-group comparisons (control and DON treatment) using the existing expression values obtained from samples. Gene expression levels were normalized as reads per kilobase of transcript per million mapped reads, and the differentially expressed genes (up- or downregulated) between the control (time zero) and the sampling points (1, 3, and 5 h after DON addition) were presented as fold-changes (**Supplementary Table 1**).

### Quantitative Reverse Transcriptase Polymerase Chain Reactions

One microgram of DNase-treated total RNA (the same sample submitted for the RNA-Seq analysis) was reverse transcribed to complementary DNA and amplified with gene-specific primers (**Supplementary Table 2**) using iTaq Universal SYBR green Supermix (Bio-Rad) on a ViiA^™^ 7 Real-Time PCR System (Applied Biosystems by Life Technologies, Austin, TX, USA). Samples were normalized to the expression of bacterial 16S rRNA methyltransferase using the ∆∆C_t_ method (Livak and Schmittgen, 2001; Cikos et al., 2007). These analyses were conducted in triplicate.

### Statistical Analysis

For bacterial cell counts, DON concentrations, and quantitative polymerase chain reaction (qPCR) analysis of transcripts, each sample was analyzed in triplicate, and mean values were determined. The relevant reduction of DON was calculated as follows:

Reduction of DON concentration (%) = (CDON added – CDON residual)/CDON added × 100.

The collected data were analyzed using SAS (SAS for Windows, Version 9.1, SAS institute, Cary, NC, USA) or Sigmaplot 12.5 (Systat Software, Inc). The data were tested for normality using the Kolmogorov–Smirnov method and equal variance (*P* value to reject was set for 0.05). Multiple group comparisons of normally distributed data were conducted by one-way analysis of variance, followed by Fisher’s protected least significant difference test. Treatments were arranged in a completely randomized fashion.

## Results

### High Concentrations of Deoxynivalenol Affect *D. mutans* 17-2-E-8 Growth and Its Ability to Transform Deoxynivalenol Into 3-*epi*-Deoxynivalenol

**Figure 1:**
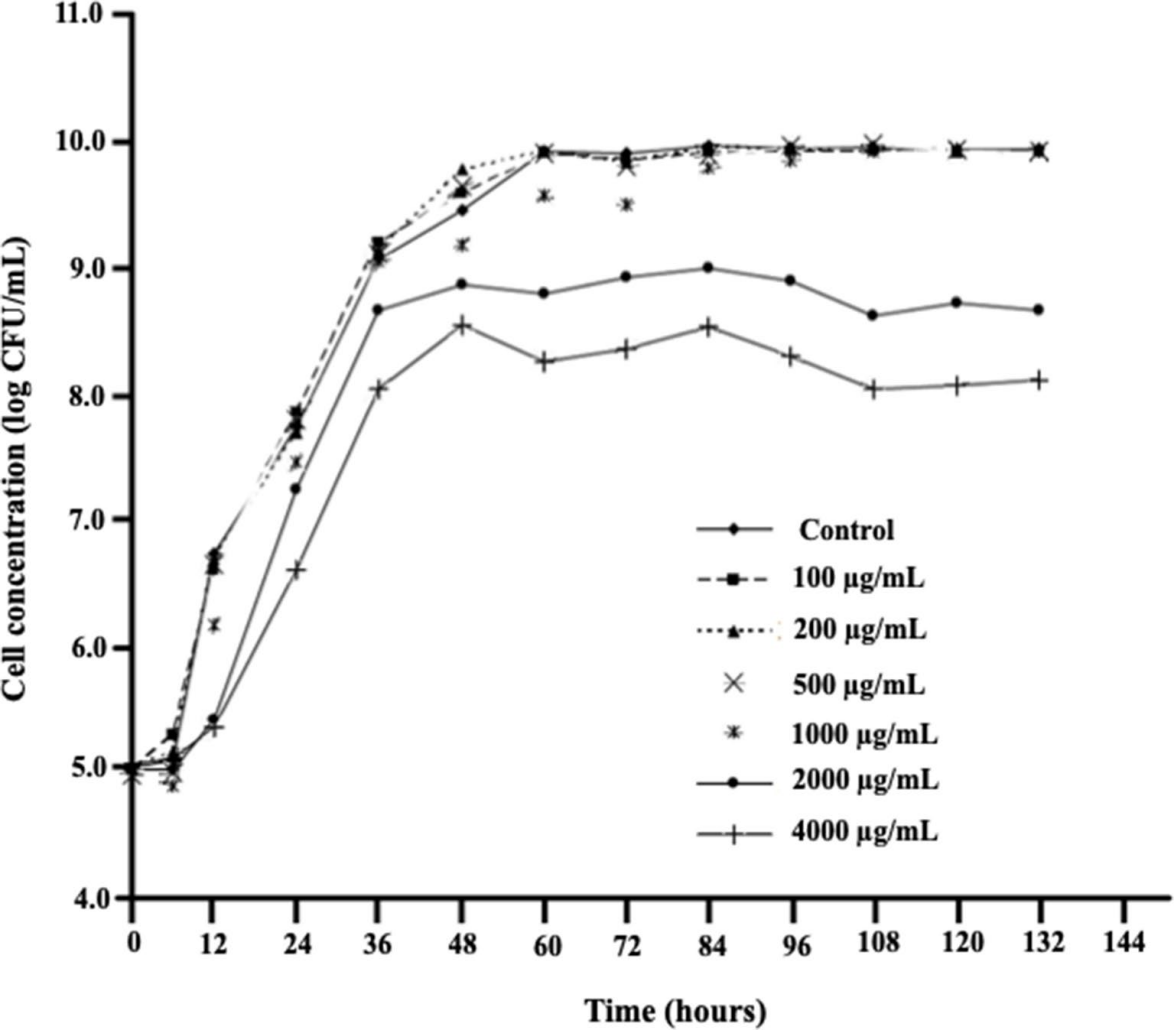
The effect of increasing DON concentrations on growth patterns of *D. mutans* 17-2-E-8 in cornmeal broth cultures at 28°C and 200 rpm. The suppressive effect of DON was only noticed in concentrations that surpassed the 1,000 µg/ml indicating a very effective defense mechanism(s) of this bacterium against DON. The protected least significant difference [PLSD_(0.05)_] of the presented results was 0.32.

*D. mutans* 17-2-E-8 cells exhibited similar growth rates when grown in the complete absence or presence of low- to medium-range concentrations of DON (100 to 500 μg/ml) (**Figure 1**) indicating an effective adaptive/response mechanism(s), whereas growth was negatively affected only by high DON concentrations (2,000–4,000 μg/ml) (**Figure 1**). The reported high levels of DON exceed the maximum tolerated levels of DON in human food chain (2 μg/ml) and animal feed chain (5 μg/ml) by at least 400–2,000 times. Interestingly, the addition of 1,000 μg/ml DON resulted in a reduced growth only within the first 72 h following media inoculation, indicating that *D. mutans* 17-2-E-8 cells in the stationary phase may be more resistant to DON compared with actively dividing/growing cells.

**Figure 2:**
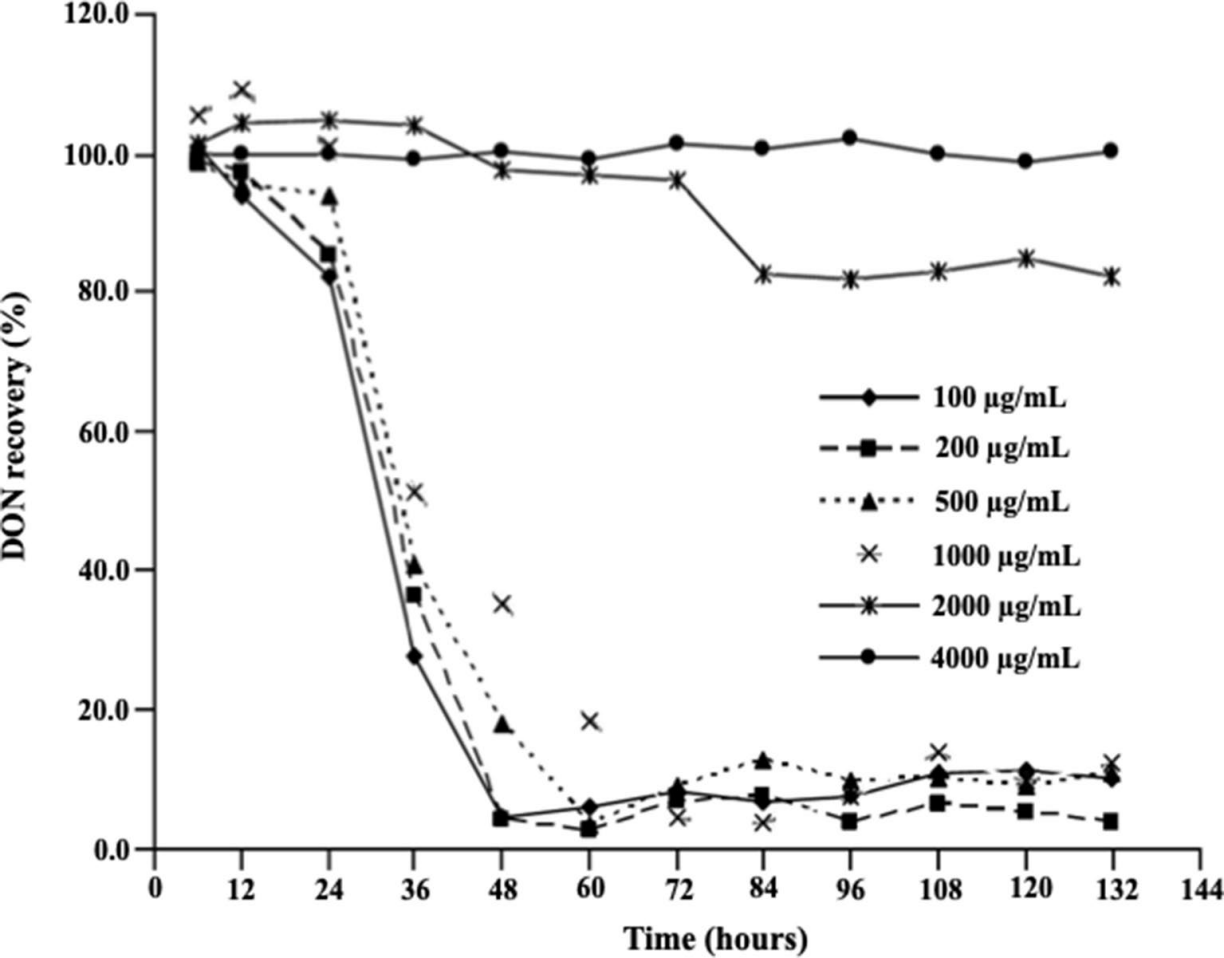
Deoxynivalenol biotransformation capacity of *D. mutans* 17-2-E-8. The ability of *D. mutans* 17-2-E-8 to transform DON (in growth media) is only saturated at extremely high concentrations of the toxin. Concentrations that ranged from 10 to 1,000 µg/ml had no negative effect on the bacterium’s ability to oxidize/epimerize DON (He et al., 2016) in cornmeal broth cultures at 28°C and 200 rpm. The protected least significant difference [PLSD_(0.05)_] of the test was 4.0.

**Figure 2** demonstrates the effect of DON concentrations on the ability of *D. mutans* 17-2-E-8 to metabolize DON, which has been previously shown to occur through the formation of 3-*epi*-DON using 3-keto-DON as an intermediate (Hassan et al., 2017). As expected, a reduction in DON concentrations was observed in cultures incubated with up to 1,000 µg/ml DON in parallel to our earlier reported studies (He et al., 2016; Hassan et al., 2017). This is despite the fact that a DON concentration of 1,000 µg/ml decreased the bacterial transformation rates of this mycotoxin in comparison with lower DON concentrations (in the 100–500 µg/ml range) within the 36–72-h time frame (**Figure 2**). Increasing DON concentrations beyond that point significantly and negatively correlated with the ability of *D. mutans* 17-2-E-8 to eliminate DON from the growth medium. The inhibitory effect of high DON concentrations on the transformation functionality was most evident at the 4,000 μg/ml, where the transformation activity was completely abrogated and could not be restored even with extended incubation times, compared with the 2,000 μg/ml dose, where the DON transformation capacity was restored partially by 84 h of incubation (**Figure 2**). This latter observation might be partially connected with the reduced sensitivity of bacterial cells (in their stationary phase) toward DON, resulting in an increased cell viability reported earlier, around the same time frame.

### Deoxynivalenol Increased Multiple Defense Mechanism(s) Involved in the Bacterial Response Toward Xenobiotics and Toxins

To identify *D. mutans* 17-2-E-8 genes/enzymes that might play a role in this microbe response to DON, we profiled it's transcriptome over multiple time points after challenging overnight-grown *D. mutans* 17-2-E-8 cultures with 50 μg/ml DON.

**Figure 3:**
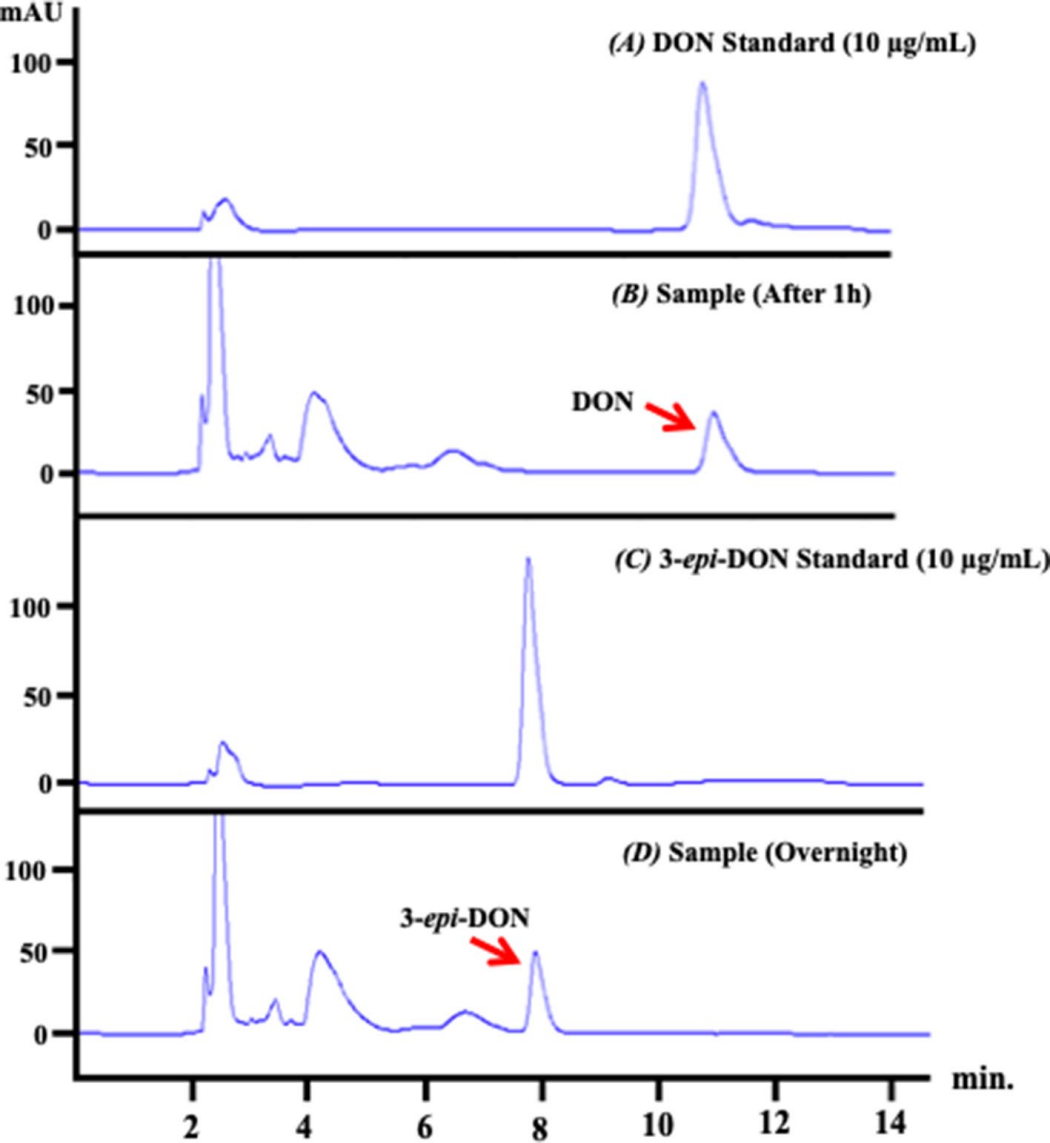
*D. mutans* 17-2-E-8 ability to biotransform DON. The obtained high-performance liquid chromatography results of overnight bacterial culture samples confirmed the ability of *D. mutans* 17-2-E-8 to epimerize DON and form the 3-*epi*-DON stereoisomer. The bacterial culture was utilized directly for total RNA preparation. Panel **(A)** represents DON standard in LB broth while panel **(B)** represents the fresh bacterial culture supplemented with DON (50 µg/ml) after one hour of growth. Panel **(C)** reflects the chromatogram of 3-epi-DON standard in fresh LB broth while the **(D)** panel shows the depleted bacterial culture after an overnight incubation with DON confirming DON disappearance and complete transformation into 3-*epi*-DON.

The analysis of the depleted bacterial cultures confirmed DON reduction and its transformation to 3-*epi*-DON (**Figure 3**) as expected (Hassan et al., 2017). DON disappearance from the analyzed samples, coupled with 3-*epi*-DON accumulation as a final product, is particularly evident in **Figure 3-D**.

Transcriptional profiling of *D. mutans* 17-2-E-8 exposed to DON revealed 433 transcripts that were upregulated (≥threefold) within 5 h of DON addition (maximum response). The results are listed in **Supplementary Table 1**, along with the microbe’s response after 1 and 3 h. While this study focused solely on the upregulated transcripts (bearing in mind potential future industrial applications) rather than the downregulated ones, we have listed all downregulated and upregulated transcripts alike in **Supplementary Table 1** due to their scientific importance.

The close inspection of the upregulated transcripts revealed the induction of several key stress and resistance genes/proteins that included, but not limited to, multiple adenosine 5’-triphosphate-binding cassette (ABC) transporters, ribosome-associated translation inhibitors, and porins in addition to various enzymes that could be implicated in xenobiotics detoxification(s) such as dehydrogenases, dioxygenases, a cytochrome P450, a chloramphenicol phosphotransferase, and a glutathione S-transferase. A short list of selected proteins previously reported to be part of microbial defense mechanisms (Pasternak et al., 1989; Niederweis, 2003; Harland et al., 2005; Piddock, 2006), coupled with their fold increases, is shown in **Table 1**. The majority of the upregulated transcripts within the 5-h exposure period belonged to the ABC-dependent transporters/effluxors (45%, 32 out of 71) with increases that spanned 3.04-fold (JP74_19625 ABC transporter permease) to ∞ (JP74_23460 sugar ABC transporter permease). The second protein group/response mechanism that was upregulated by large was cellular dehydrogenases (15%) and oxidoreductase (14%) (**Table 1**). The importance of some of these dehydrogenases [especially pyrroloquinoline quinone (PQQ)-dependent ones] is discussed later. Finally, multiple coping enzymes/proteins, including a cytochrome P450 (3.01-fold increase), a glutathione S-transferase (3.01-fold increase), a ribosome-associated translation inhibitor (7.9-fold increase), the JP74_12500 porin (3.24-fold increase), and a chloramphenicol phosphotransferase (3.15-fold increase), reflected increased upregulation in expression levels (**Table 1**).

**Table 1:** *Devosia mutans* 17-2-E-8 genes related to detoxification/defense mechanism(s) that were upregulated due to deoxynivalenol (50 µg/ml) inclusion in bacterial growth media.

Locus tag		Predicted function		Fold increase (1 h)		Fold increase (3 h)		Fold increase (5 h)		
JP74_23460		Sugar ABC transporter permease		1		1		∞		
JP74_18865		PQQ-dependent dehydrogenase/DepA		2.02		4.34		15.25		
JP74_15915		PQQ-dependent dehydrogenase		2.86		7.41		13.78		
JP74_14225		Ribosome-associated translation inhibitor RaiA		3.37		3.58		7.9		
JP74_19085		Amino acid ABC transporter substrate-binding protein		1.72		2.21		6.53		
JP74_16245		Dihydrolipoamide dehydrogenase		2.32		5.44		6.23		
JP74_16255		Branched-chain alpha-keto acid dehydrogenase		2.96		5.6		6.13		
JP74_16260		Pyruvate dehydrogenase		2.41		5.09		5.46		
JP74_03245		Pyrroloquinoline quinone biosynthesis protein PqqD		3.64		4.01		5.15		
JP74_03255		Pyrroloquinoline quinone biosynthesis protein PqqB		3		5.56		5.03		
JP74_16575		Lactoylglutathione lyase/glyoxalase/bleomycin resistance protein		4.57		3.31		4.74		
JP74_16765		Chemotaxis protein		3.15		2.68		4.52		
JP74_20580		A PQQ-dependent dehydrogenase		2.1		2.42		4.4		
JP74_06865		Peptide ABC transporter substrate-binding protein		1.76		2.93		4.35		
JP74_15220		peptide ABC transporter substrate-binding protein		1.55		2.84		4.21		
JP74_10080		ABC transporter substrate-binding protein		2.58		1.76		4.17		
JP74_12385		Basic membrane lipoprotein Med, periplasmic binding		1.64		1.99		4.11		
JP74_05670		EamA-like transporter		3.27		2.67		4.1		
JP74_07645		NADPH-dependent FMN reductase		3.81		3.38		3.98		
JP74_03700		3,4-dihydroxyphenylacetate 2,3-dioxygenase		2.59		2.22		3.96		
JP74_17605		ABC transporter permease		4.1		2.22		3.94		
JP74_11190		Oxidoreductase		1.77		2.4		3.9		
JP74_09830		Peptide ABC transporter permease		2.22		1.86		3.88		
JP74_16760		Chemotaxis protein CheY		2.34		2.76		3.86		
JP74_22840		NAD-dependent dehydrogenase		2.44		2.34		3.77		
JP74_12230		Magnesium transporter		3.17		2.37		3.75		
JP74_22190		Multidrug ABC transporter ATP-binding protein		3.16		1.82		3.75		
JP74_12310		Glutamine ABC transporter permease		2.77		2.04		3.75		
JP74_05630		ABC transporter permease		3		2.67		3.69		
JP74_22940		Aldo/keto reductase		2.41		1.83		3.62		
JP74_04995		Succinate:quinone oxidoreductase		2.01		2.2		3.54		
JP74_06225		ABC transporter		2.84		1.66		3.46		
JP74_19390		ABC transporter ATP-binding protein		3.2		2.21		3.44		
JP74_14455		ABC transporter ATP-binding protein		2.01		1.63		3.43		
JP74_20255		Multidrug ABC transporter ATP-binding protein		1.62		1.49		3.43		
JP74_21500		ABC transporter		3.26		1.26		3.34		
JP74_14870		ABC transporter permease		3.2		1.6		3.33		
JP74_17530		Oxidoreductase		2.83		1.6		3.29		
JP74_03240		Pyrroloquinoline quinone biosynthesis protein PqqE		1.91		3.02		3.27		
JP74_01045		ABC transporter permease		2.28		1.78		3.27		
JP74_12500		Porin		1.68		1.34		3.24		
JP74_06190		Aldo/keto reductase		2.36		2.05		3.23		
JP74_19630		ABC transporter permease		3.02		1.78		3.23		
JP74_00365		ABC transporter		3.66		2.14		3.23		
JP74_00025		ABC transporter		2.62		1.64		3.23		
JP74_10990		ABC transporter permease		2.67		1.43		3.22		
JP74_12660		Multidrug transporter		2.53		1.6		3.22		
JP74_17025		Multidrug transporter		3.05		1.71		3.22		
JP74_03590		3-ketoacyl-ACP reductase		2.81		1.92		3.21		
JP74_20625		Alcohol dehydrogenase		2.79		2.35		3.21		
JP74_12350		Transporter		2.62		1.97		3.19		
JP74_04990		Succinate:quinone oxidoreductase		2.27		2.07		3.18		
JP74_14500		Oxidoreductase		2.53		1.73		3.18		
JP74_08035		Ketol-acid reductoisomerase		3.39		3.37		3.17		
JP74_14235		ABC transporter		1.98		1.63		3.15		
JP74_19500		Chloramphenicol phosphotransferase		1.86		1.8		3.15		
JP74_05635		Sulfonate ABC transporter ATP-binding protein		2.31		1.98		3.15		
JP74_22160		ABC-type antimicrobial peptide transport		2.41		1.87		3.14		
JP74_09880		Oxidoreductase		2.48		1.84		3.14		
JP74_00740		ABC transporter		2.17		1.85		3.14		
JP74_21490		ABC transporter permease		3.14		1.63		3.13		
	JP74_18605		3-hydroxy-2-methylbutyryl-CoA dehydrogenase		2.36		1.79		3.13	
	JP74_08775		ABC transporter		2.84		1.43		3.09	
	JP74_20595		ABC transporter		2.85		2.19		3.09	
	JP74_12305		ABC transporter		2.45		1.97		3.09	
	JP74_20565		FAD/FMN-containing dehydrogenase		2.4		1.85		3.05	
	JP74_19625		ABC transporter permease		2.65		1.86		3.04	
	JP74_17675		Alkanesulfonate monooxygenase		2.22		1.64		3.02	
	JP74_19870		Cytochrome P450		2.08		1.88		3.01	
	JP74_02475		Glutathione S-transferase		2.03		1.85		3.01	
	JP74_05835		Alcohol dehydrogenase		1.93		2.29		3	

### The Transcription of Specific Pyrroloquinoline Quinone-Dependent Pathways/Dehydrogenases Was Evidently Upregulated in Response to Deoxynivalenol

**Figure 4:**
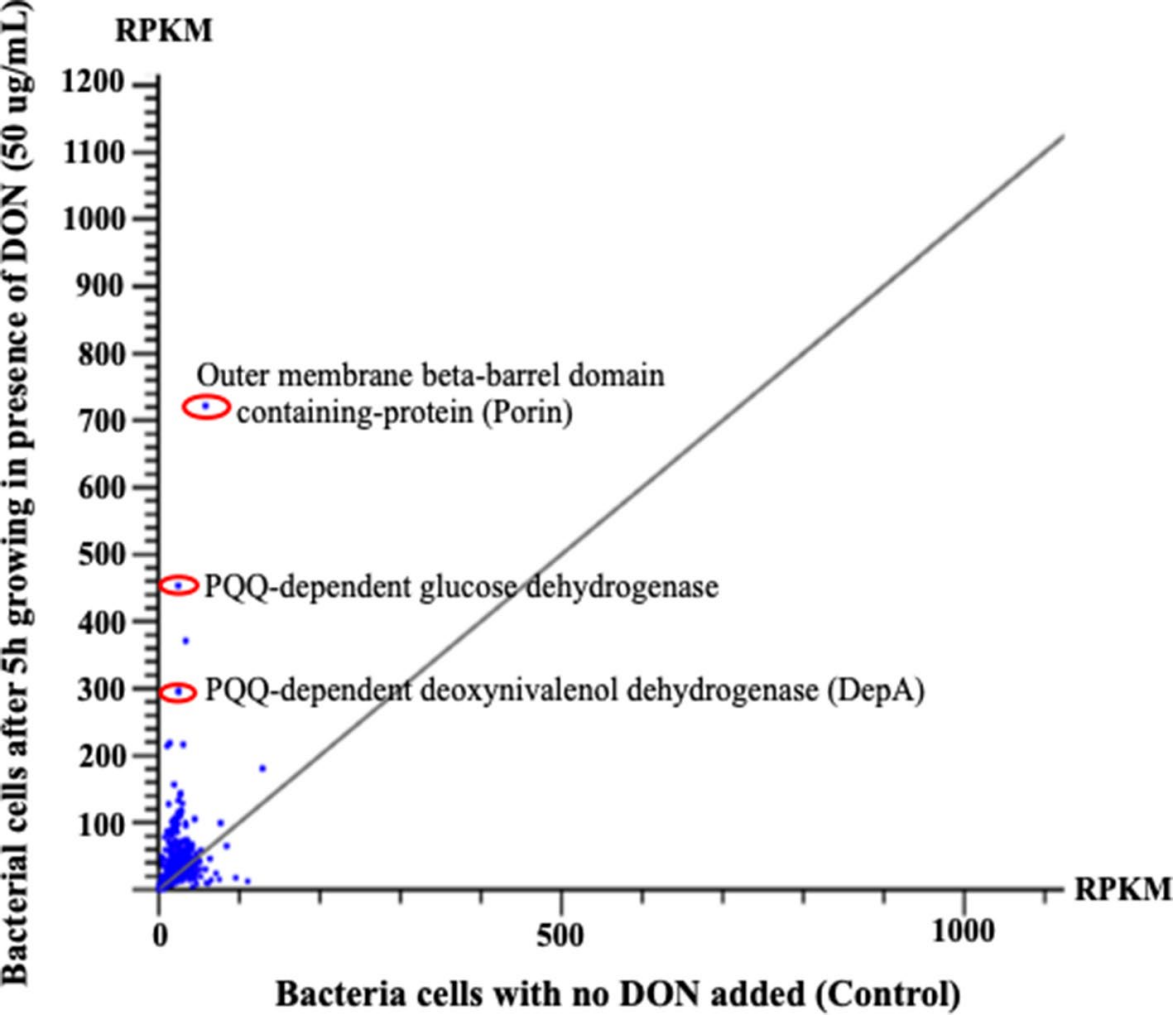
The horizontal comparisons of expression levels of RNA samples extracted from bacterial cells before and after challenging with DON (50 µg/ml). The graph clearly shows that multiple PQQ-dependent enzymes/defense mechanisms have increased due to DON treatment. The presented results were normalized using the reads per kilobase of transcript per million mapped reads. Points/dots above the diagonal gray line indicate upregulated transcripts, while points beneath that line indicate the downregulation/suppression of the involved transcript.

Notably many PQQ-dependent transcripts were overexpressed as a result of DON inclusion within the growth medium (**Figure 4** and **Table 1**). Multiple PQQ-dependent dehydrogenases including JP74_20580, JP74_15915, and JP74_18865 were upregulated up to 4.4-, 13.78-, and 15.25-fold increases, respectively, after 5-h growth in DON-supplemented medium, in comparison with the negative control (**Table 1**). At the same time, enzymes putatively involved in PQQ redox cofactor biosynthesis, such as PqqB, PqqD, and PqqE, each showed a gradual increase in expression levels spanning the 1–3-h time frame, with a maximum induction (5.03-, 5.15-, and 3.27-fold, respectively) after 5 h of continuous DON exposure (**Table 1**).

**Figure 5:**
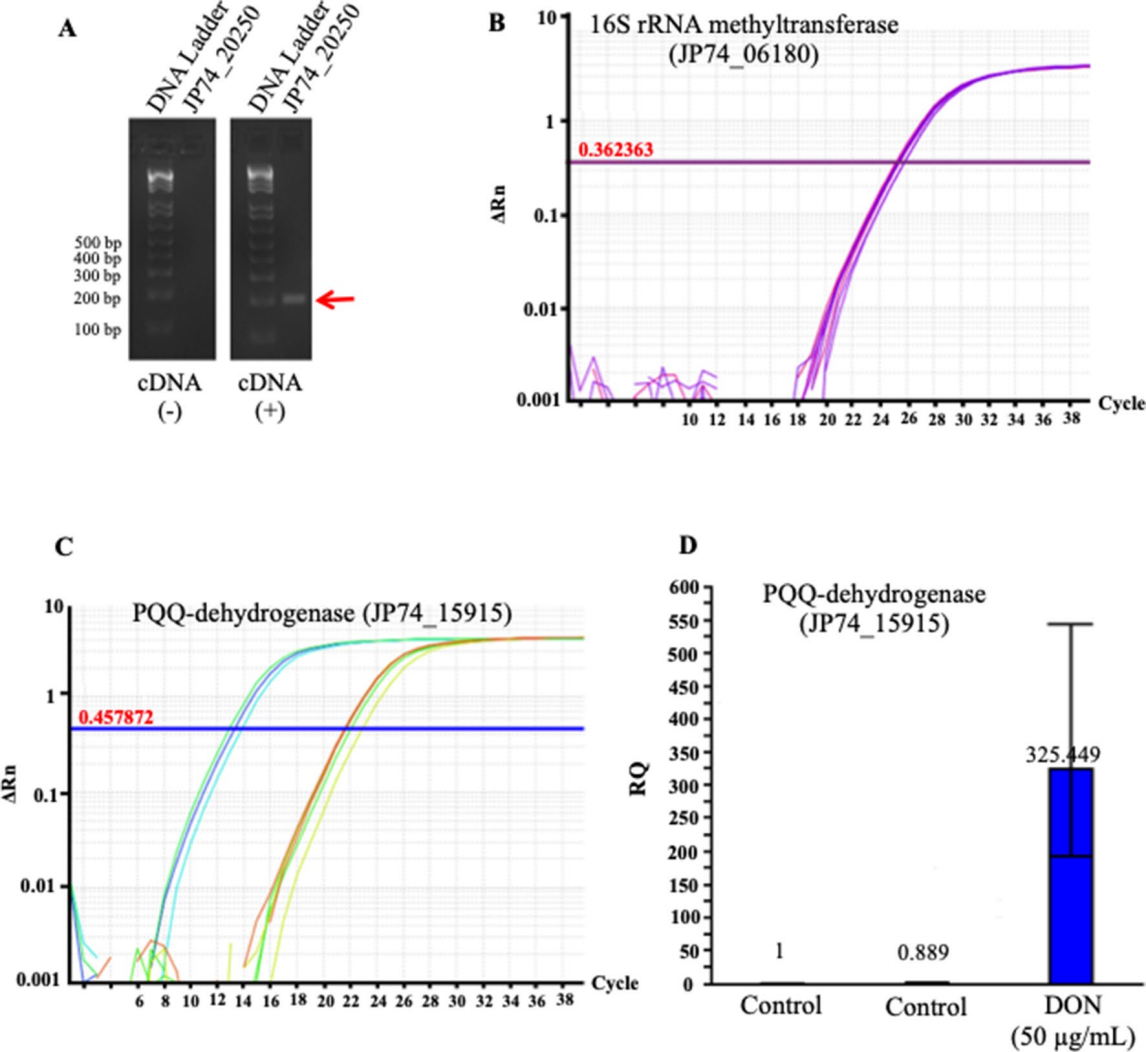
The tendencies observed in the RNA_Seq results were confirmed using a quantitative and semiquantitative polymerase chain reactions (qPCR): **(A)** the semiquantitative detection/amplification of the JP74_20250 gene to check the quality of the prepared complementary DNA strands, **(B)** the 16S rRNA methyltransferase (JP74_06180) gene was used as the housekeeping gene for the normalization and calculation of fold increases of transcripts amplified using the qPCR approach, **(C)** an example of the qPCR amplification/detection of a PQQ-dependent dehydrogenase (JP74_15915) and its relative expression in response to DON **(D)** in comparison to unchallenged bacterial cells (control).

To validate these results, we used the qPCR screening to verify the expression levels of several genes, including two PQQ-dependent dehydrogenases (JP74_18865 and JP74_15915), an oxidoreductase (JP74_11190), and a hypothetical protein (JP74_20250). A close correlation was observed between the expression levels investigated by the qPCR approach and the obtained RNA-Seq data, thereby substantiating the RNA-Seq outcomes (**Figure 5**). Surprisingly, the qPCR data reflected higher fold increases in some cases (**Figure 5D**) in comparison with the results collected by RNA-Seq. Such a nonconformity between the two methodologies is well known and has been previously reported (Robinson et al., 2015; Everaert et al., 2017).

## Discussion

Understanding how mycotoxins influence prokaryotes began to emerge as an important area of future research focus (Robert et al., 2017; Liao et al., 2018). This is particularly true in the light of the recent studies that implicated gut microbiota in DON-induced anorexia (Peng et al., 2017b), DON-induced immunotoxicity with reported changes in gut bacteria (Liao et al., 2018), and DON ability to modulate the genotoxicity of colibactin-producing *Escherichia coli* strains/isolates (Payros et al., 2017). The ability of even a single dose of mycotoxins to induce significant changes within the gut microbial communities (Ishikawa et al., 2017) establishes the urgency to fully engage in deciphering the effect of DON on as many prokaryotic species as possible in order to delineate the production and health-related ramifications of DON/microbial interactions.

The presented work is among the first to look into the changes induced in the global gene-regulation/expression levels (as a result of DON exposure) within a potent bacterial strain isolated mainly for its ability to transform/detoxify DON (He et al., 2016). While the studied bacterial isolate, *D. mutans* 17-2-E-8, did not originate from gut, the recently established role of *Devosia* species in gut’s health with links associating the decreased abundance of this genus with irritable bowel syndrome in humans (Ng et al., 2013) and gut microbiota dysbiosis (due to *Salmonella infantis* infections) in animals both justify looking into any transcriptomic changes induced in this genus due to DON exposure. Furthermore, the ability of multiple *Devosia* strains including *D. mutans* 17-2-E-8 (He et al., 2016), *D.* sp. ANSB714 (Zhao et al., 2016), *D.* RS1 and SS5 (Sato et al., 2012), and *D.* sp. strain A16 (Yin et al., 2016) to transform DON to less toxic metabolites, either 3-keto-DON and/or 3-*epi*-DON (He et al., 2015a; Hassan et al., 2016), with the possibility of incorporating of such detoxification pathways into animal feed applications (Yin et al., 2016; Zhao et al., 2016), necessitates a closer engagement with this genus to avoid any uncounted for detrimental effects in the future.

The collected results clearly show that the studied bacterium (pure culture) has the ability to withstand much higher concentrations of DON (up to 1,000 μg/ml), which fall significantly above the maximum permitted levels of DON (5–10 μg/ml) within any animal feed/human food. This in turn enhances the chances for incorporating the actively growing cells of this microorganism in future DON-mitigation strategies intended for the animal feed chain. At the same time, the influence of DON on *D. mutans* 17-2-E-8 needs to be further complemented with data related to mixed cultures and/or bio-niches that mimic the composition of natural microbiota within the animal gut, where other factors such as pH, low-oxygen levels, and bacterial competition(s) play fundamental roles and possibly render *D. mutans* 17-2-E-8 more/less susceptible toward DON.

The obtained results reflect a three-legged strategy that this bacterium utilizes to circumvent DON toxicity: (a) first, by upregulating multiple ABC-dependent membrane transporters and efflux pumps that remove many undesirable toxins/chemicals (including DON) from the cellular environment (Sharma et al., 2003; Barabote et al., 2011); (b) second, the bacterium engages a toxin-specific detoxification mechanism carried by certain dehydrogenases/oxidoreductases to reduce and eliminate the cellular toxicity of the involved toxin. In DON case, the 17-2-E-8 isolate specifically upregulates a PQQ-dependent glucose dehydrogenase that oxidizes DON to 3-keto-DON as a first-step detoxification followed by the subsequent reduction to the more stable/nontoxic 3-*epi*-DON isomer (Carere et al., 2018a; Hassan et al., 2017). The very recent isolation of the DON-C3 carbon oxidation/reduction enzymes using various protein fractionation techniques (Carere et al., 2018a; Carere et al., 2018b) supports this conclusion; (c) third, this microorganism employs an arsenal of auxiliary proteins including porins, glutathione S-transferases, ribosome-associated translation inhibitors, and chloramphenicol phosphotransferases, which is part of an efficient strategy that aims to block/minimize the entry of DON to the cytoplasm, introduce chemical modifications to minimize the overall toxicity, reduces translation errors/inhibitions (Agafonov and Spirin, 2004) associated with DON exposure (Pan et al., 2014), and/or increase toxin’s solubility to enhance toxin’s efflux/elimination. Although some of the mechanisms identified earlier are confined to DON-transforming *Devosia* (the PQQ-dependent pathway for example), the other mechanisms that are built upon the upregulation of adenosine 5’-triphosphate-dependent transporters are more universal in nature and indeed representative of widely speared response/resistance mechanisms in bacteria including multiple antibiotic resistance (Nikaido, 2009; Nikaido and Pages, 2012; Du et al., 2015; Wendlandt et al., 2015).

While our results are consistent with the ones reported by Park et al. (2014) in which the authors showed the ability of DON to influence ABC-dependent membrane transporters, bacterial chemotaxis, succinate dehydrogenases, and enzymes related to the glyoxylate cycle within *E. coli* K-12, they differ in the observed tendencies and magnitudes. The low concentrations that were used in Park’s study (0.2 and 2 µg/ml) did not lead to any upregulations of identified ABC transporters, while the upregulation of such transporters was certainly evident in our case. This can be attributed to the higher concentrations of DON that we have used (50 µg/ml). The facts that most prokaryotes/eukaryotes show a significant increase in a large number of ABC transporters in the due course of many mycotoxins exposures (Anzai et al., 2010; Dubey et al., 2014; Walter et al., 2015) validate our results and show the importance of this defense mechanism within its evolutionary context. In addition, the fact that DON levels can reach as high as 25–93 µg/g in plant materials and wheat kernels (Sinha and Savard, 1997) makes the use of high doses of DON (50 µg/ml in our RNA_Seq studies) more relevant to certain scenarios of animal/human exposure.

Finally, the results of this study can be useful not only in understanding how gram-negative bacteria respond to DON at the transcriptomic level but can also aid in developing and optimizing DON detoxification approaches (through the identification of novel enzymes) that can alleviate the toxicity of DON and possibly be utilized in a later stage within refined agricultural and industrial applications (Carere et al., 2018a).

## Author Contributions

Design of the work: YH, JH, DL, and TZ. Conducting experiments: YH and JH. Interpretation of data and analysis: YH, JH, DL, and TZ. Drafting the work: YH, JH, and TZ. Final approval: YH, JH, DL, and TZ.

## Conflict of Interest Statement

The authors declare that the research was conducted in the absence of any commercial or financial relationships that could be construed as a potential conflict of interest.

## Supplementary Material

Supplementary Table 1 A comprehensive list of *Devosia mutans* 17-2-E-8 transcripts that have been modulated (up or down-regulated) in response to deoxynivalenol (50 µg/ml) inclusion in bacterial growth media.
