# Supplementary material for: Understanding the Bacterial Response to Mycotoxins: The Transcriptomic Analysis of Deoxynivalenol-Induced Changes in Devosia mutans 17-2-E-8
Source: Front Pharmacol. 2019 Nov 14;10:1098. doi: 10.3389/fphar.2019.01098 (PMC6868067; doi:10.3389/fphar.2019.01098)
Supplement: Supplementary file 2 [file Table_2.docx]

**Supplementary Table (2).** cDNA primers used to confirm the upregulation of selected RNA transcripts as a result for deoxynivalenol (50 ug/mL) inclusion in *Devosia mutans* 17-2-E-8 growth media.

| **Sequence** | **Transcript** |
| --- | --- |
| 5`-GAAGTCGAGGATATGGATGCC-3` | gfo/Idh/MocA family oxidoreductase (JP74_11190) |
| 5`-TCAACCTCACCTATCGCAAC-3` | gfo/Idh/MocA family oxidoreductase (JP74_11190) |
| 5`-AGGTGCATTCAGAATCCCAG-3` | DepA (JP74_18865) |
| 5`-TCTGGCTTCGGTTGCTATG-3` | DepA (JP74_18865) |
| 5`-GCATACCAATACCCCAGTAGG-3` | Pyrrolo-quinoline quinone (JP74_15915) |
| 5`-AGATCGTGTGGGAAAAGCAG-3` | Pyrrolo-quinoline quinone (JP74_15915) |
| 5`-TGGAACGAGGTCAAGGTTTC-3` | Hypothetical protein (JP74_20250) |
| 5`-GGATAGAGATCGGAGTTCACC-3` | Hypothetical protein (JP74_20250) |
| 5`-GCCTTCTGCACGACATAGTC-3` | 16S rRNA methyltransferase (JP74_06180) |
| 5`-AAGTCATCCTCTTCAACGGC-3` | 16S rRNA methyltransferase (JP74_06180) |
