# Supplementary figures and images for: Understanding the Bacterial Response to Mycotoxins: The Transcriptomic Analysis of Deoxynivalenol-Induced Changes in Devosia mutans 17-2-E-8
Source: Front Pharmacol. 2019 Nov 14;10:1098. doi: 10.3389/fphar.2019.01098 (PMC6868067; doi:10.3389/fphar.2019.01098)

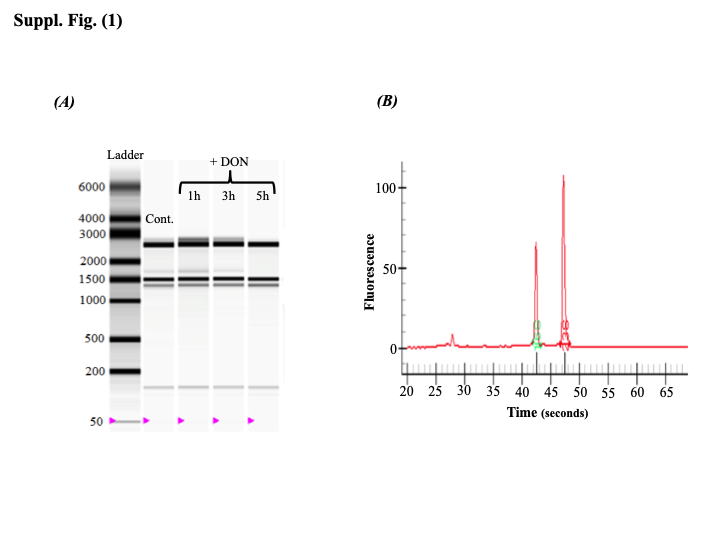

Supplement: Supplementary file 3 [file Image_1.tiff]
